# Structured Allocation of Transcatheter Aortic Valve Replacement Patients during Coronavirus Disease 2019 Pandemic: Impact on Patient Selection and Clinical Results

**DOI:** 10.3390/jcdd9060189

**Published:** 2022-06-10

**Authors:** Nora Berisha, Kathrin Klein, Verena Veulemans, Oliver Maier, Kerstin Piayda, Stephan Binnebößel, Shazia Afzal, Amin Polzin, Ralf Westenfeld, Patrick Horn, Christian Jung, Malte Kelm, Christine Quast, Tobias Zeus

**Affiliations:** 1 Department of Cardiology, Pulmonology and Vascular Medicine, Medical Faculty, Heinrich Heine University, Moorenstr. 5, 40225 Düsseldorf, Germany; nora.berisha@med.uni-duesseldorf.de (N.B.); kathrin.klein@med.uni-duesseldorf.de (K.K.); verena.veulemans@med.uni-duesseldorf.de (V.V.); oliver.maier@med.uni-duesseldorf.de (O.M.); kerstin.piayda@med.uni-duesseldorf.de (K.P.); stephan.binneboessel@med.uni-duesseldorf.de (S.B.); shazia.afzal@med.uni-duesseldorf.de (S.A.); amin.polzin@med.uni-duesseldorf.de (A.P.); ralf.westenfeld@med.uni-duesseldorf.de (R.W.); patrick.horn@med.uni-duesseldorf.de (P.H.); christian.jung@med.uni-duesseldorf.de (C.J.); malte.kelm@med.uni-duesseldorf.de (M.K.); christine.quast@med.uni-duesseldorf.de (C.Q.); 2 CARID (Cardiovascular Research Institute Düsseldorf), Moorenstr. 5, 40225 Düsseldorf, Germany

**Keywords:** TAVR, COVID-19, aortic stenosis, allocation algorithm

## Abstract

Due to shortages of medical resources during the Coronavirus Disease 2019 (COVID-19) pandemic, an allocation algorithm for Transcatheter Aortic Valve Replacement (TAVR) was established. We investigated the impact on patient selection and procedural results. In total, 456 TAVR patients before (pre-COVID-19 group) and 456 TAVR patients after (COVID-19 group) the implementation of our allocation algorithm were compared. Concerning patient characteristics, the COVID-19 group revealed a higher rate of cardiac decompensations/cardiogenic shocks (10.5% vs. 1.3%; *p* < 0.001), severe angina pectoris (Canadian Cardiovascular Society (CCS) II, III and IV: 18.7% vs. 11.8%; *p* = 0.004), troponin elevation (>14 ng/L: 84.9% vs. 77%; *p* = 0.003) and reduced left ventricular ejection fraction (LVEF) (<45%: 18.9% vs. 12%; *p* = 0.006). Referring to procedural characteristics, more predilatations (46.3% vs. 35.1%; *p* = 0.001) and a longer procedural time (80.2 min (+/−29.4) vs. 66.9 min (+/−17.5); *p* < 0.001) were observed. The success rate was evenly high; no differences in safety parameters were reported. Examining the utilization of hospital resources, the COVID-19 group showed a shorter in-hospital stay (8.4 days (+/−5.9) vs. 9.5 days (+/−9.33); *p* = 0.041) and fewer TAVR patients were treated per month (39 (+/−4.55) vs. 46.11 (+/−7.57); *p* = 0.03). Our allocation algorithm supported prioritization of sicker patients with similar efficient and safe TAVR procedures. In-hospital stay could be shortened.

## 1. Introduction

Transcatheter Aortic Valve Replacement (TAVR) is a well-established procedure for the treatment of severe aortic valve stenosis [1,2,3]. Starting as an alternative for older and sicker patients who, due to their serious comorbidities, would have been considered as inoperable and not suitable for surgical aortic valve replacement (SAVR) [4,5], it is now an established therapeutic option for even intermediate and low-risk surgical candidates [6]. The infrastructure of heart centers around the world has adopted to the fast-growing need for TAVR options and successful TAVR programs have been created.

With the outbreak of the Coronavirus Disease 2019 (COVID-19) pandemic, healthcare systems all over the world had to restructure and reorganize their non-COVID-19 care because of limited medical capacities and resources as well as the risk of COVID-19 spread, also affecting TAVR treatment. Every institution had to define their way of fulfilling the needs of COVID-19 care and non-COVID-19 care. Medical societies helped to define allocation algorithms, and this led to a restructuring of many TAVR programs [7,8,9,10,11,12,13]. Our heart center started early with the development of an internal allocation algorithm with the aim of offering resources to the patients with urgent need and the ambition of defining patient cohorts who could go on a waiting list without unrecognized deterioration of their disease. This algorithm was based on the American College of Cardiology/Society for Cardiovascular Angiography and Interventions (ACC/SCAI) [14] and European Society of Cardiology (ESC) [8] guidelines for TAVR treatment during the pandemic and was discussed and approved by the local Corona ethics committee.

Until now, it has not been shown whether an algorithm considering ACC/SCAI [14] as well as ESC [8] recommendations for aortic valve stenosis and TAVR management during COVID-19 effectively changed patient characteristics of a TAVR cohort. Further questions were whether the procedure itself could be performed with similar safety and efficacy as compared to prepandemic interventions and whether differences during in-hospital follow-up occurred.

## 2. Materials and Methods

### 2.1. Study Population

**Figure 1:**
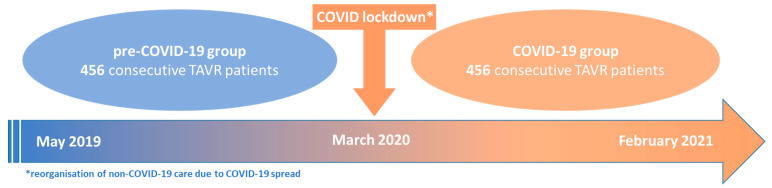
Consort.

We investigated two patient cohorts: 456 consecutive patients that underwent TAVR procedure under non-pandemic circumstances from 19 May 2019 to 15 March 2020 formed the pre-COVID-19 group, while the pandemic group (COVID-19 group) included 456 consecutive patients who received TAVR after the implementation of our allocation algorithm between 16 March 2020 and 22 February 2021 (Figure 1).

The justifying indication and suitability for TAVR treatment was discussed in our regularly held multidisciplinary heart team meetings.

As a guidance for TAVR patient selection and treatment timing, the ACC/SCAI [14] triage considerations and ESC [8] guidelines were used to establish an allocation algorithm (Table 1). Depending on the in-hospital and 30-day mortality estimation, we categorized and prioritized the patients scheduled for TAVR into three groups, each one combined with a timing suggestion. Patients were either classified as requiring “emergency/urgent TAVR” within several days (category 1), classified as category 2, indicating that, due to their “urgent but stable” status, TAVR should be performed within four weeks or classified as category 3, considering TAVR as an “elective” procedure that can be postponed to an undefined date. During the pandemic, we followed this algorithm and our decision making was based on the degree of urgency TAVR treatment was needed.

### 2.2. Preprocedural Period

Our study population underwent preprocedural standard diagnostic work up, including, among others, transthoracic echocardiography.

### 2.3. Procedure

The TAVR procedure followed a routine protocol and was performed in a hybrid operating room by the heart team. The access site was either transfemoral or transapical. All transfemoral cases were done under conscious sedation. The valve-type choice remained at the discretion of the interventional cardiologist (Edwards Sapien, Medtronic Evolut or Boston Acurate Neo) as well as the evaluation whether pre- and/or postdilatation was needed.

The observed and compared intraprocedural events incorporated the Valve Academic Research Consortium (VARC)-2 criteria [19].

### 2.4. Postprocedural Period

In our postprocedural examination, we compared our study groups in aspects of postinterventional transthoracic echocardiography, blood analysis and postprocedural events that occurred in-hospital according to the VARC-2 criteria [19].

### 2.5. Statistical Analysis

For continuous variables, we verified the fulfilment of normal distribution and presented them by mean +/− standard deviation. Comparisons were performed assessing the unpaired student’s *t*-test. Categorial data are listed as frequencies and percentages. For the examination of differences, we used the chi-square test. All statistical tests were two-sided, with a *p*-value < 0.05 considered as statistically significant. All statistical data analyses were performed using SPSS.

## 3. Results

### 3.1. Baseline Characteristics

The average age was 80.8 (+/−6.8) years in the pre-COVID-19 group and 81.2 (+/−5.8) years in the COVID-19 group (Table 2 “baseline parameters”).

Except for the higher percentage of peripheral artery disease in the pre-COVID-19 group (pre-COVID-19: 25.2% vs. COVID-19: 20.3%; *p* < 0.001), the comparison did not reveal significant differences concerning the analyzed comorbidities (Table 3 “preexisting illnesses/comorbidities”).

When taking the cardiac history and cardiac symptoms (Table 4 “cardiac anamnesis/history”) into consideration, multiple significant differences were found. There was a higher rate of cardiac decompensations during the last 12 months (pre-COVID-19: 14.5% vs. COVID-19: 24.5%; *p* < 0.001), as well as cardiac decompensations/cardiogenic shocks (pre-COVID-19: 1.3% vs. COVID-19: 10.5%; *p* < 0.001) in the COVID-19 group.

**Figure 2:**
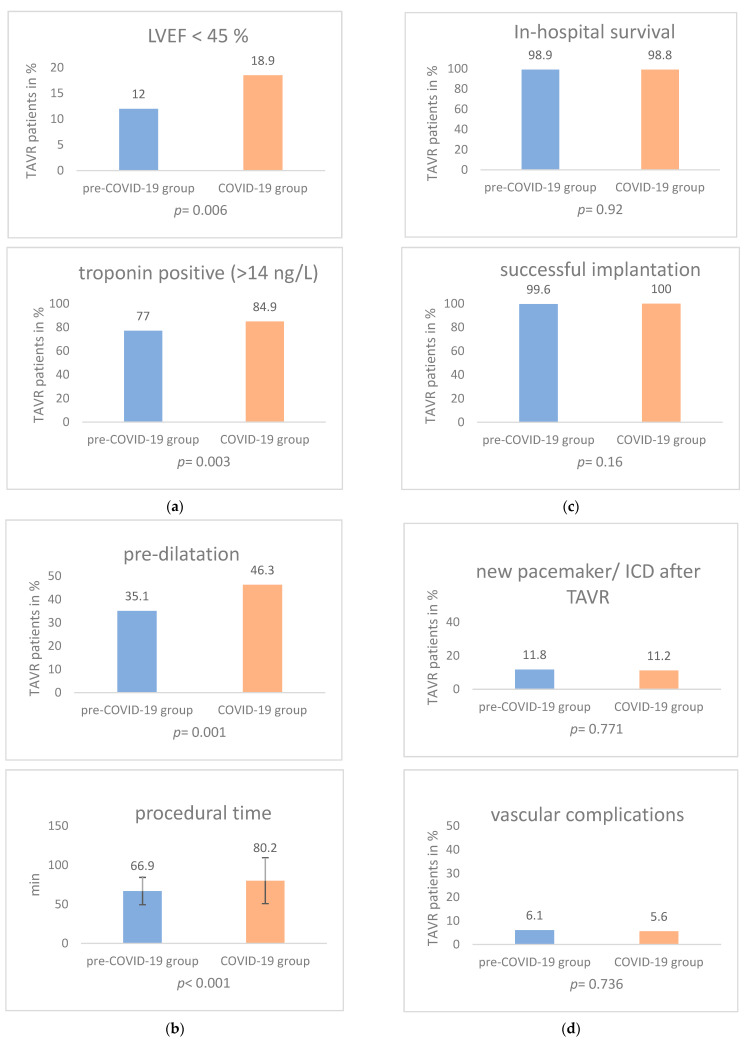
(**a**) Patient characteristics; (**b**) procedural characteristics; (**c**) efficacy and survival; (**d**) VARC-2 criteria.

The COVID-19 group showed more conduction abnormalities, such as left bundle branch block (LBBB) (pre-COVID-19: 4.8% vs. COVID-19: 10.9%; *p* = 0.001) and right bundle branch block (RBBB) (pre-COVID-19: 5.3% vs. COVID-19: 8.9%; *p* = 0.036). Furthermore, the percentage of patients reaching Canadian Cardiovascular Society (CCS) classification score II, III and IV was significantly higher in the COVID-19 group (pre-COVID-19: 11.8% vs. COVID-19: 18.7%; *p* = 0.004). In addition, troponin elevation could be measured more frequently in the COVID-19 group (troponin level above 14 ng/L: pre-COVID-19: 77% vs. COVID-19: 84.9%; *p* = 0.003, Table 5 “laboratory”; Figure 2a). Further analysis of other baseline characteristics and variables related to patients’ clinical data (listed in Table 2, Table 3, Table 4 and Table 5) did not exhibit significant differences.

### 3.2. Preprocedural Echocardiography and Computed Tomography

In Table 6, echocardiographic parameters are depicted. The percentage of patients with a reduced left ventricular ejection fraction was significantly higher in the COVID-19 group (LVEF below 45%: pre-COVID-19: 12% vs. COVID-19: 18.9%; *p* = 0.006; Figure 2a).

### 3.3. Procedure

The TAVR procedure in the COVID-19 group was characterized by a higher rate of predilatations (pre-COVID-19: 35.1% vs. COVID-19: 46.3%; *p* = 0.001; Figure 2b) and a longer procedural time (pre-COVID-19: 66.9 min (+/−17.5) vs. COVID-19: 80.2 min (+/−29.4); *p* < 0.001; Figure 2b).

Further procedural details are displayed in Table 7 “procedural details” and Table 8 “intraprocedural complications”.

### 3.4. Postprocedural Data and In-Hospital Follow-Up

Patients undergoing TAVR in the COVID-19-period had a significantly shorter length of in-hospital stay (pre-COVID-19: 9.5 days (+/−9.33) vs. COVID-19: 8.4 days (+/−5.9); *p* = 0.041; Table 9 “postprocedural data and follow-up”). The COVID-19 group continued to show LVEF impairment (LVEF below 45%) after the TAVR procedure (pre-COVID-19: 7.8% vs. COVID-19: 13.1%; *p* = 0.014). Furthermore, the total number of patients receiving TAVR per month was significantly lower during COVID-19 (pre-COVID-19: 46.11 (+/−7.57) vs. COVID-19: 39 (+/−4.55); *p* = 0.03). The other investigated postprocedural criteria, involving VARC-2 [19] parameters, can be seen in Table 9 “postprocedural data and follow-up”, Table 10 “outcome parameters post-TAVR” and Figure 2c,d.

## 4. Discussion

The COVID-19 pandemic had a severe impact on healthcare systems all around the world. Heart centers, like other medical facilities, had to adapt and restructure, also affecting Transcatheter Aortic Valve Replacement (TAVR). For the first time, our analysis investigated the effects of an allocation algorithm based on the pandemic-related TAVR management recommendations published by the American College of Cardiology/Society for Cardiovascular Angiography and Interventions (ACC/SCAI) [14] and the European Society of Cardiology (ESC) [8].

Our main results are:

1. The implementation of our allocation prioritized sicker patients for TAVR during the pandemic;
2. The TAVR procedure showed similar success, despite the higher complexity in the COVID-19 group;
3. TAVR during COVID-19 could be performed with a shorter in-hospital stay and similar in-hospital safety results.

### 4.1. Patient Prioritization through Our Allocation Algorithm

Concerning patient characteristics, the COVID-19 group significantly differed from pre-COVID-19 group. The subjects who received TAVR in the COVID-19-era showed cardiac decompensations/cardiogenic shocks, conduction abnormalities, severely symptomatic angina pectoris, troponin elevation and reduced left ventricular ejection fraction at a significant higher rate. This could be assigned to the effective implementation of our allocation algorithm.

Thus, TAVR treatment in the pandemic was primarily performed in patients who, due to their critical cardiac constitution, inevitably needed Transcatheter Aortic Valve Replacement and could not tolerate treatment postponement (category 1 and 2 of our allocation algorithm). This was achieved despite limited non-COVID care resources because of mainly two factors: the postponement of TAVR procedure in category 3 triaged patients and the reduction of TAVR procedures performed per month. The fact that the European system for cardiac operative risk evaluation (EuroSCORE) [21] does not reflect the precise clinical condition in sufficient detail, but also incorporates extracardial factors, explains why there is no significant difference in that aspect between our compared patient groups. The pre-COVID-19 group, as an example, suffers from peripheral artery disease at a higher percentage (pre-COVID-19: 25.2% vs. COVID-19: 20.3%; *p* <0.001). This counterbalances the higher LVEF impairment of the COVID-19 group (LVEF below 45%: pre-COVID-19: 12% vs. COVID-19: 18.9%; *p* = 0.006), as both parameters affect the EuroSCORE [21].

Other publications also mentioned structured allocation in context of their patient selection for TAVR treatment [22,23]. However, results differed because, firstly, they focused on the ACC/SCAI [14] triage recommendations only and, secondly, they seemed to have incorporated a less strict interpretation of them [22]. The latter was discussed by the authors themselves. The COVID-19 group in the Oxford experience [22] appeared healthier in certain preprocedural parameters than the pre-COVID-19 group. TAVR patients during the pandemic, as an example, were significantly younger (“pre-COVID-19: 82 years (+/−6) vs. COVID-19: 79 years (+/−7); *p* < 0.01”) and were characterized by a lower EuroScore II (“pre-COVID-19: 4.6 (3.0, 9.0) vs. COVID-19: 3.1 (1.8, 5.4); *p* = 0.01”).

Valdebenito et al., in their study [9], mentioned the ACC/SCAI [14] guidelines when thematizing patient selection during COVID-19, but did not observe significant differences in the preprocedural characteristics of their pre-COVID-19 and their COVID-19 patient group. The proof for their prioritization and selection concept being successfully implemented, thus, remains questionable.

In a Dutch single-center analysis [10], a classification system was created for patient prioritization requiring TAVR during COVID-19, which the authors characterized as “largely comparable with the later published ACC/SCAI consensus statement”. Whether this classification evoked any significant differences to TAVR treatment under regular healthcare circumstances cannot be analyzed due to the fact that only patients who underwent TAVR during COVID-19 were evaluated.

Amgad Mentias et al. [24], in their publication, proposed an algorithm for timing of a planned TAVR procedure; however, in contrast to our allocation algorithm, they did not refer to the ESC [8] guidelines. Additionally, a comparison between patients receiving TAVR before and during COVID-19 was not established by them.

### 4.2. Higher Complexity of TAVR Procedures during COVID-19

Looking at the procedure itself, safety and procedural success did not differ significantly. Differences could be observed in aspects of procedure time and usage of intraprocedural predilatation. This may be attributed mainly to two factors. First, the COVID-19 group was sicker with respect to ejection fraction and cardiogenic decompensation/shock. Therefore, more sophisticated periprocedural anesthesiologic care was needed. Second, during the pandemic, the Boston Acurate Neo valve, which requires mandatory predilatation, was implanted more frequently. This predilatation has a negative impact on the procedure time.

### 4.3. Safe and Effective TAVR Treatment during COVID-19 with a Shorter In-Hospital Stay and Similar In-Hospital Results

In our analysis, the COVID-19 group showed a shorter in-hospital stay with a potential risk reduction of COVID-19 transmission and viral exposure as a result, also achieved by intermediate care (IMC) and intensive care unit (ICU) capacity savings. In our preprocedural COVID-19 testing during the pandemic, two COVID-19 positive patients could be identified, resulting in a TAVR treatment postponement. The shorter in-hospital stay, as well as the recognition that we did not provoke a worsening of the patients’ outcome, supported the safety of our triage concept in the pandemic.

Interestingly, the incidence of new onset of left bundle branch block was higher in the COVID-19 group. However, the pacemaker implantation rate was not affected by this change in electrical conductance. The follow-up has to take this observation into careful consideration.

In our in-hospital follow-up period, the Valve Academic Research Consortium VARC-2 [19] criteria showed comparable results to other TAVR cohorts which have been treated during the pandemic [9,22].

## 5. Conclusions

Our analysis proved the implementation of an allocation algorithm as effective, with the result that TAVR treatment was primarily concentrated on patients at the most critical cardiac condition. The TAVR procedure itself showed similar success rates, despite higher complexity in the COVID-19 group. In-hospital stay was shorter in the COVID-19 group and in-hospital follow-up results did not differ significantly between the COVID-19 and the pre-COVID-19 group.

## 6. Limitations

Some limitations should be considered when evaluating and interpreting our study. This was a single-center, retrospective, non-randomized and observational study. There may exist confounders and a selection bias.

## Figures and Tables

**Table 1:** Allocation algorithm.

1. Emergency/Urgent	2. Urgent but Stable	3. Elective
- Estimated 30-day mortality approximately 30% [15,16,17,18] - Immediate treatment during in-hospital course	- Estimated 30-day mortality approximately 8.4% [15,16,17,18] - TAVR treatment within four weeks	- Estimated 30-day mortality approximately 4.3% [15,16,17,18] - TAVR treatment postponement with careful outpatient follow-up
- Symptomatic severe to critical aortic stenosis - Cardiogenic shock/cardiac decompensations - NYHA III and IV - CCS II, III and IV - Heart failure associated with LVEF impairment	- Mildly symptomatic severe aortic stenosis - NYHA I and II - CCS 0 and I	- Asymptomatic severe aortic stenosis - All patients not meeting category 1 and 2

**Table 2:** Baseline parameters.

	Pre-COVID-19 (*n* = 456)	COVID-19 (*n* = 456)	*p*-Value
Body Mass Index (BMI) (kg/m^2^ +/− SD)	26.5 (+/−5)	26.8 (+/−4.9)	0.499
female	222 (48.7%)	196 (43.2%)	0.095
age (years +/− SD)	80.8 (+/−6.8)	81.2 (+/−5.8)	0.367
EuroSCORE (+/− SD)	10.7 (+/−2.4)	10.8 (+/−2.3)	0.665

Continuous data are presented as the means +/− standard deviation (SD); categorial data are listed as numbers (*n*) with percentages (%).

**Table 3:** Preexisting illnesses/comorbidities.

	Pre-COVID-19 (*n* = 456)	COVID-19 (*n* = 456)	*p*-Value
arterial hypertension	409 (89.7%)	376 (87.9%)	0.385
chronic obstructive pulmonary disease (COPD)	114 (25%)	126 (29.4%)	0.138
cerebrovascular disease	66 (14.5%)	80 (18.7%)	0.091
peripheral artery disease	115 (25.2%)	87 (20.3%)	<0.001
diabetes mellitus	125 (27.4%)	134 (31.3%)	0.203
frailty	67 (14.7%)	48 (11.2%)	0.124
porcelain aorta	34 (7.5%)	21 (4.9%)	0.117
previous cerebrovascular accident (stroke)/transient ischemic attack (TIA)	59 (12.9%)	59 (13.8%)	0.701

All data are listed as numbers (*n*) with percentages (%).

**Table 4:** Cardiac anamnesis/history.

	Pre-COVID-19 (*n* = 456)	COVID-19 (*n* = 456)	*p*-Value
coronary heart disease	336 (73.7%)	349 (76.5%)	0.357
previous percutaneous coronary intervention (PCI)	175 (38.4%)	178 (41.6%)	0.330
previous pacemaker/implantable cardioverter defibrillator (ICD) implantation	65 (14.3%)	59 (13.8%)	0.84
New York Heart Association (NYHA) classification Score II, III and IV	443 (97.1%)	418 (97.7%)	0.631
Canadian Cardiovascular Society (CCS) classification Score II, III and IV	54 (11.8%)	80 (18.7%)	0.004
left bundle branch block (LBBB)	22 (4.8%)	44 (10.9%)	0.001
right bundle branch block (RBBB)	24 (5.3%)	36 (8.9%)	0.036
previous cardiac decompensation (in last 12 months)	66 (14.5%)	105 (24.5%)	<0.001
cardiogenic shock/cardiac decompensation	6 (1.3%)	45 (10.5%)	<0.001
cardiopulmonary resuscitation	5 (1.1%)	7 (1.6%)	0.489

All data are listed as numbers (*n*) with percentages (%).

**Table 5:** Laboratory.

	Pre-COVID-19 (*n* = 456)	COVID-19 (*n* = 456)	*p*-Value
troponin positive (>14 ng/L)	351 (77%)	355 (84.9%)	0.003
glomerular filtration rate (GFR) (mL/min +/− SD)	57.2 (+/−21.3)	58.8 (+/−20.9)	0.356
hemoglobin (g/dl +/− SD)	12.3 (+/−1.7)	12.4 (+/−1.7)	0.144

Continuous data are presented as the means +/− standard deviation (SD); categorial data are listed as numbers (*n*) with percentages (%).

**Table 6:** Preprocedural echocardiographic characteristics.

	Pre-COVID-19 (*n* = 456)	COVID-19 (*n* = 456)	*p*-Value
maximum pressure gradient across the aortic valve (AV maxPG) (mmHg +/− SD)	62.2 (+/−24.8)	64.6 (+/−23.3)	0.152
mean pressure gradient across the aortic valve (AV meanPG) (mmHg +/− SD)	38.1 (+/−16.4)	39.4 (+/−15.4)	0.227
LVEF below 45%	53 (12%)	78 (18.9%)	0.006

Continuous data are presented as the means +/− standard deviation (SD); categorial data are listed as numbers (*n*) with percentages (%).

**Table 7:** Procedural details.

	Pre-COVID-19 (*n* = 456)	COVID-19 (*n* = 456)	*p*-Value
femoral access site	447 (98%)	446 (98.5%)	0.623
valve in valve	19 (4.2%)	9 (2.1%)	0.080
Valve type			
- Medtronic Evolut	361 (79.2%)	310 (72.6%)	0.022
- Edwards Sapien	83 (18.2%)	94 (22%)	0.157
- Boston Acurate Neo	12 (2.6%)	23 (5.4%)	0.036
predilatation	160 (35.1%)	198 (46.3%)	0.001
fluoroscopy time (min +/− SD)	18.21 (+/−8.1)	18.7 (+/−8.9)	0.391
cumulative dose (cGy*cm^2^ +/− SD)	3995.9 (+/−3582.4)	4275.7 (+/−3781.2)	0.261
amount of radioactive contrast medium (mL +/− SD)	83.6 (+/−38.9)	87.5 (+/−41.6)	0.155
procedural time (min +/− SD)	66.9 (+/−17.5)	80.2 (+/−29.4)	<0.001
postdilatation	57 (12.5%)	58 (13.6%)	0.642
paravalvular leak			
-no	293 (64.2%)	270 (64.4%)	0.955
-mild	159 (34.9%)	147 (35.1%)	0.947
-moderate	4 (0.9%)	2 (0.5%)	0.474

Continuous data are presented as the means +/− standard deviation (SD); categorial data are listed as numbers (*n*) with percentages (%).

**Table 8:** Intraprocedural complications.

	Pre-COVID-19 (*n* = 456)	COVID-19 (*n* = 456)	*p*-Value
conversion to open surgery	2 (0.4%)	0 (0%)	0.170
cardiopulmonary resuscitation	3 (0.7%)	3 (0.7%)	0.938
mechanical circulatory support	2 (0.4%)	1 (0.2%)	0.6
aortic dissection	0 (0%)	0 (0%)	
cardiac tamponade	1 (0.2%)	1 (0.2%)	0.964
vascular complications	28 (6.1%)	24 (5.6%)	0.736
ventricular perforation	0 (0.0%)	1 (0.2%)	0.302
major bleeding	4 (0.9%)	3 (0.7%)	0.768
life-threatening/disabling bleeding	6 (0.2%)	2 (0.5%)	0.183

All data are listed as numbers (*n*) with percentages (%).

**Table 9:** Postprocedural data and follow-up.

	Pre-COVID-19 (*n* = 456)	COVID-19 (*n* = 456)	*p*-Value
in-hospital stay (day +/− SD)	9.5 (+/−9.33)	8.4 (+/−5.9)	0.041
TAVR patients per month	46.11 (+/−7.57)	39 (+/−4.55)	0.03
sepsis	3 (0.7%)	1 (0.2%)	0.348
endocarditis	0 (0%)	2 (0.5%)	0.144
myocardial infarction type 1 [20]	0 (0%)	0 (0%)	
acute kidney injury	53 (11.6%)	60 (14.1%)	0.28
maximum pressure gradient across the aortic valve (AV maxPG) (mmHg +/− SD)	15.4 (+/−8.5)	15.4 (+/−8.3)	0.958
mean pressure gradient across the aortic valve (AV meanPG) (mmHg +/− SD)	8.2 (+/−4.5)	8.5 (+/−4.5)	0.386
left ventricular ejection fraction (LVEF) below 45%	31 (7.8%)	52 (13.1%)	0.014

Continuous data are presented as the means +/− standard deviation (SD); categorial data are listed as numbers (*n*) with percentages (%).

**Table 10:** Outcome parameters post-TAVR.

	Pre-COVID-19 (*n* = 456)	COVID-19 (*n* = 456)	*p*-Value
30-day mortality	5 (1.1%)	5 (1.2%)	0.92
transient ischemic attack (TIA) (<30 d)	4 (0.9%)	6 (1.4%)	0.461
stroke (<30 d)	9 (2%)	12 (2.8%)	0.418
new left bundle branch block (LBBB)	34 (7.5%)	61 (14.3%)	0.001
new pacemaker/implantable cardioverter defibrillator (ICD) implantation	54 (11.8%)	48 (11.2%)	0.771

Categorial data are listed as numbers (*n*) with percentages (%).

## Data Availability

Data and materials are available on demand from the corresponding author.
